# Detection of Cognitive Performance Deterioration Due to Cold-Air Exposure in Females Using Wearable Electrodermal Activity and Electrocardiogram

**DOI:** 10.3390/bios15020078

**Published:** 2025-01-29

**Authors:** Youngsun Kong, Riley McNaboe, Md Billal Hossain, Hugo F. Posada-Quintero, Krystina Diaz, Ki H. Chon, Jeffrey Bolkhovsky

**Affiliations:** 1 Biomedical Engineering, College of Engineering, University of Connecticut, Storrs, CT 06269, USA; riley.mcnaboe@uconn.edu (R.M.); hugo.posada-quintero@uconn.edu (H.F.P.-Q.); ki.chon@uconn.edu (K.H.C.); 2 Analog Devices, Wilmington, MA 01887, USA; mdbillal.hossain@analog.com; 3 Leidos, Reston, VA 20190, USA; krystina.l.diaz.ctr@health.mil; 4 Naval Submarine Medical Research Laboratory, Groton, CT 06349, USA; jeffrey.b.bolkhovsky.civ@health.mil

**Keywords:** electrodermal activity, heart rate variability, machine learning, cold stress, cognitive performance, reaction time

## Abstract

Prolonged exposure to cold air can impair reaction time and cognitive function, which can lead to serious consequences. One mitigation strategy is to develop models that can predict cognitive performance by tracking physiological metrics associated with cold stress. As females are evidenced to be more sensitive to cold exposure, this study investigated the relationship between physiological metrics and cognitive performance deterioration of female subjects under cold stress. Wearable electrodermal activity (EDA) and electrocardiogram (ECG) were collected from nineteen females who underwent five sessions of a cognitive task battery—assessing reaction time, memory, and attention—in a cold (10 °C) environment. Machine learning classifiers showed higher cognitive performance classification accuracies with heart rate variability (HRV) features than with EDA features. Particularly in detecting performance deterioration in a task associated with assessing short-term memory, our support vector machine classifier with HRV features showed an 82.4% accuracy, with a sensitivity of 84.2% and a specificity of 80.6%, whereas a 55.4% accuracy with a sensitivity of 44.7% and a specificity of 66.7% was obtained with EDA features. Our results demonstrate the feasibility of detecting performance deterioration from females who underwent cold exposure using wearable EDA and ECG, allowing for preventive measures to reduce risk in cold environments, especially for female military personnel.

## 1. Introduction

Certain occupations, such as aircraft pilots, athletes, and military personnel, demand prompt and accurate decision-making, requiring individuals to perform at their best under challenging circumstances. However, exposure to cold air can significantly impair individual reaction time and cognitive function [1,2,3]. Prolonged exposure to cold air can lead to deterioration of occupational performance, possibly to the extent of causing serious accidents or fatalities in operational environments especially in diving, construction, and military operations in cold weather. Therefore, it is essential to detect performance deterioration early, prior to the occurrence of any related/associated hazards. Wearable monitors that track cognitive performance would be of significant benefit to divers, construction workers, and military personnel. These devices would facilitate prompt notifications to users, their colleagues, and supervisors in the event of detected cognitive performance deterioration. This, in turn, enables the implementation of preemptive interventions (e.g., the consumption of high-calorie snacks, the automatic deployment of heating devices, and others).

Males and females have differences in body composition, autonomic mechanisms, hormonal activity, and other factors that can affect cognitive performance during cold exposure. For example, females tend to have more fat mass and less lean mass, and vice versa in males [4]. In addition, males tend to have a relatively greater cardiac sympathetic tone and reduced cardiac parasympathetic tone in response to cold exposure compared to females [5,6,7]. In addition, females produce more estrogen than males, which increases vasodilation and consequently blood flow to the skin [8]. These can affect thermoregulation and thermogenesis mechanism during cold exposures, resulting in physiological and cognitive performance differences between males and females. Studies have also shown that females are more sensitive to cold exposure and have shivering effects earlier than males [9,10]. A recent study exhibited the negative effect of 10 °C cold air exposure on cognitive performance in females, but not in males [7]. Therefore, the current study focuses on the unique physiological effects or features that may arise in cold-stressed female subjects.

When humans are exposed to cold air, their bodies undergo physiological changes as the body employs adaptive mechanisms to preserve and generate body heat to maintain core temperature [11]. Such changes include vasoconstriction and shivering thermogenesis [12], which are associated with the autonomic nervous system (ANS). The ANS helps maintain homeostasis of the body, including regulation of heart rate, blood pressure, and body temperature [13]. The sympathetic nervous system (SNS), a branch of the ANS associated with stimulation of bodily functions, activates vasoconstriction in the peripheral blood vessels, reducing blood flow to the skin and other superficial tissues, which decreases heat loss [14]. Studies have also shown activity increases in the parasympathetic nervous system (PNS), the branch of ANS often associated with adaptation/habituation, after the initial SNS response and with cold habituation [15,16,17,18].

These ANS activities can be assessed using heart rate variability (HRV) and electrodermal activity (EDA) data, which can be collected using wearable devices that employ either electrocardiography (ECG) or photoplethysmography (PPG) sensors. HRV is a noninvasive measure for assessing the dynamics of the ANS [19]. Because HRV is straightforward to compute from ECG, it has been widely used to understand the physiological effects of cold environment exposure in human subjects [17,20]. Moreover, several studies have shown that HRV can be effectively used to assess cognitive functions [21,22,23]. For example, recent studies have used HRV to detect and quantify workloads and cognitive performance degradation due to prolonged sleep deprivation in human subjects [24,25,26].

More recently, studies have shown that EDA has a high sensitivity in detecting sympathetic arousal, such as emotion, stress, and pain [27,28,29]. EDA is a measure of the skin’s electrical conductance, as the SNS activation invokes the opening of sweat gland pores [30,31]. The EDA signal is analytically processed to separate phasic and tonic components. The phasic component reflects the fast EDA dynamics (i.e., rapid changes) in response to a startle-like stimulus, while the slower tonic component (i.e., gradual changes) has been shown to be related to passive indicators of sympathetic activity, related to factors such as hydration levels and skin temperature [27]. Recent work has shown that EDA can also be analyzed in both the time and frequency domains, which captures time-varying dynamics, and is termed as the time-varying index of EDA (TVSymp). This metric has shown higher sensitivity and consistency in assessing SNS activity when compared to the phasic and tonic components of EDA [32,33,34,35]. For example, recent studies have shown that TVSymp more accurately detects cognitive performance deterioration than either the tonic or phasic EDA components [36,37].

The current study aimed to detect cold-exposed performance deterioration in females, using EDA and ECG signals collected by wearable devices. Continuous monitoring via wearable EDA and ECG devices offers a noninvasive and practical approach to detecting performance deterioration among personnel working in cold environments. ECG and EDA data were collected from participants who performed a battery of cognitive tasks while exposed to room temperature-air (23 °C) or cold-air (10 °C) environments for 140 min. Machine learning approaches were applied on features derived from ECG and EDA, features of which were subsequently assessed for the ability to detect performance degradation due to cold-air exposure in a laboratory-controlled environment.

## 2. Materials and Methods

### 2.1. Subjects

Nineteen healthy undergraduate and graduate female students (sex assigned at birth) were recruited (age: 22.7 ± 3.3; BMI: 23.0 ± 3.9) from the University of Connecticut (UConn).

### 2.2. Experiment Protocol

Subjects underwent two different temperature conditions across two separate days: room-temperature (i.e., “normal”) (23 °C) and cold-air (i.e., “cold”) (10 °C) environments. During each condition, subjects sat within a controlled laboratory environment, wore physiological monitors for the continuous collection of physiological (ECG and EDA) data, provided five minutes of baseline physiological data and then proceeded to perform five sessions of a cognitive task battery every 30 min (approximately 140 min in the cold-air environment). Note that prior studies have determined that this cold condition results in deterioration of cognitive performance and alteration in cardiac autonomic function [3,17]. The order of testing conditions (normal and cold air) was randomized. Signed consent forms were obtained before the start of each experiment.

To help control for clothing insulation across subjects, subjects wore experimenter-provided cotton clothing for each experimental day, which included socks, a long sleeve shirt, and long pants. Subjects were instructed to attach Ag/AgCl electrodes on the index and middle fingers of their non-dominant hands for EDA data collection and Ag/Cl electrodes on their chest for ECG data collection. The EDA electrodes were connected to an Empatica E4 (Boston, MA, USA) placed on subjects’ non-dominant wrist. ECG data were collected using the ScottCare Chroma Holter Monitor (ScottCare, Cleveland, OH, USA). ECG and EDA signals were collected at sampling frequencies of 256 Hz and 4 Hz, respectively. Before the electrodes were placed, the skin areas were cleaned with 70% isopropyl alcohol. Supplemental to these devices, additional ECG signals were also recorded using the Equivital EQ02+ LifeMonitor and associated chest-worn electrodes (Swavessey, UK). The core temperature of each subject was measured using an ingestible capsule sensor (VitalSense, Philips Respironics, Bend, OR, USA). Additionally, three sites of skin temperatures were collected using dermal patch sensors (VitalSense, Philips Respironics, Bend, OR, USA): the back of the dominant hand, the left calf, and the forehead. Temperature data were transmitted to the Equivital EQ02+ LifeMonitor. We calculated the area-weighted skin temperature (*T_skin_*) as follows:

(1) $$T_{skin}=0.21875\times T_{Forehead}+0.15625\times T_{hand}+0.625\times T_{calf}$$

The weights were derived from ISO 9886 [38]. As ISO 9886 provides weight-coefficients for 8 regions of skin sites, we proportionally adjusted weighting coefficients for the three skin sites (forehead, hand, and calf) to have a sum of 1 (Table A1). Finally, the mean body temperature (MBT) was calculated using Burton’s formula [39,40] as follows:

(2) $$T_{MBT}=0.36\times T_{skin}+0.64\times T_{core}$$

Table 1 summarizes the study procedures. Subjects were asked to visit the fourth floor of the Engineering and Science Building at UConn Storrs Campus on different days for each condition. Before each testing day, subjects were asked to get a minimum of 8 h of sleep and consume food no less than 3 h prior to their appointment. Additionally, they were asked to refrain from consumption of any depressants or stimulants (e.g., caffeine). On the first day, subjects completed a demographic questionnaire, including age, height, and weight. Experimenters calculated body mass index (BMI) using height and weight. Subjects were also asked to finish two practice sessions of the cognitive task battery based on the Defense Automated Neurobehavioral Assessment (DANA) to minimize the potential for learning effects. Note that we chose two practices sessions because a mild learning effect was observed during the first and second trials of DANA from which our cognitive task battery was derived [41]. Subjects completed the battery on a Galaxy Tab S7 tablet device (Samsung, Suwon, Republic of Korea) situated on a desk, while seated on a chair. Each session of the cognitive task battery, which consisted of five individual cognitive tasks, took approximately 15 min, and a cognitive task battery session was repeated every 30 min (for a total exposure duration of approximately 140 min). Therefore, subjects had 15 min of downtime in between each session. The study protocol was approved by the Institutional Review Boards at UConn and at the Naval Submarine Medical Research Laboratory (NSMRL) in Groton, CT.

### 2.3. Cognitive Performance

**Figure 1:**
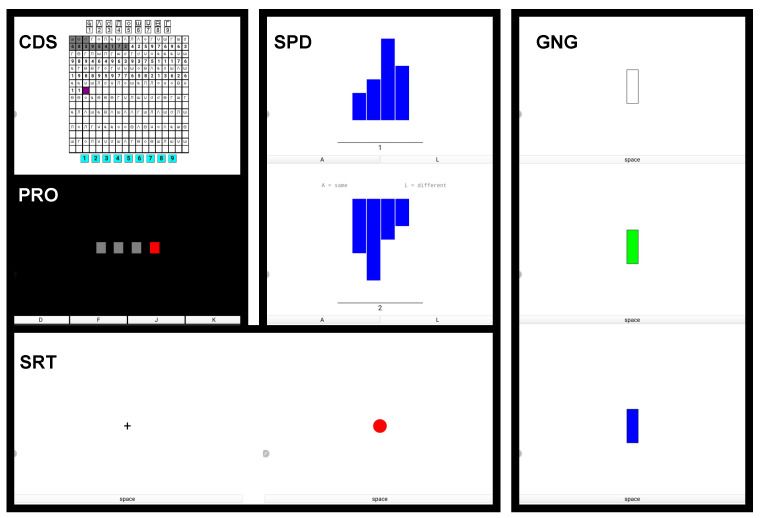
Screenshots of cognitive tasks, including code substitution (CDS), procedural reaction time (PRO), Go/No-go (GNG), spatial discrimination (SPD), and simple reaction time (SRT).

Subjects completed a battery of five cognitive tasks to measure their cognitive performance in normal and cold conditions (Table 2). The five DANA-based tasks included code substitution (CDS), procedural reaction time (PRO), Go/No-go (GNG), spatial discrimination (SPD), and simple reaction time (SRT). These tasks were selected to assess how cold exposure affects cognitive performance: short-term memory and attention (CDS), ability to accurately respond while inhibiting premature responses (PRO), omission and commission (GNG), spatial manipulation (SPD), and attention (SRT). DANA is an effective tool for comprehensive assessment of the wide range of problems that can occur during combat operations; therefore, it is an effective tool for simulating an operational environment [41]. These tasks were built using the Inquisit 6 application (Millisecond Software, LLC, Seattle, WA, USA), installed on the tablet. We measured subjects’ performance based on their reaction time to every task, because accuracies on these tasks did not show any deterioration during prolonged cold exposures. Table 2 and Figure 1 show the list of cognitive tasks and screenshots of each task, respectively.

#### 2.3.1. Code Substitution (CDS)

Subjects were shown a code of nine symbols at the top of the screen, which corresponded with the digits (1–9). Subjects were asked to press buttons (also 1–9) located at the bottom of the screen as they corresponded to the symbols in the matrix, as quickly and as many as possible. The matrix of symbols was randomized for each trial. The task evaluated the immediate memory and attention of subjects.

#### 2.3.2. Procedural Reaction Time (PRO)

Subjects were shown a horizontal row of four boxes onscreen. When a box turned red, subjects were asked to tap the corresponding buttons at the bottom of the screen as fast as possible. This task assessed their ability to accurately respond while inhibiting premature responses.

#### 2.3.3. Go/No-Go (GNG)

Subjects were shown a rectangle on the screen in either a horizontal or vertical orientation. They were then asked to press the space bar button at the bottom of the screen as fast as possible if the rectangle turned green, but not to respond if the rectangle turned blue. This task assessed the ability of subjects to control responses in making omissions and commissions.

#### 2.3.4. Spatial Discrimination (SPD)

Subjects were shown a set of two histograms, with each histogram consisting of four bars. The second histogram, presented after the first set, was rotated by either 0°, 90°, 180°, or 270° degrees. The subjects were required to determine whether the two sets of histograms were congruent. The task evaluated spatial manipulation abilities.

#### 2.3.5. Simple Reaction Time (SRT)

Subjects were instructed to tap in the bottom of the screen as soon as a red circle appeared. The task took approximately 90 s to complete and was designed to assess the subjects’ vigilant attention. The task evaluated the participants’ ability to sustain attention and quickly respond to stimuli.

### 2.4. Definition of Deteriorated Performance

**Figure 2:**
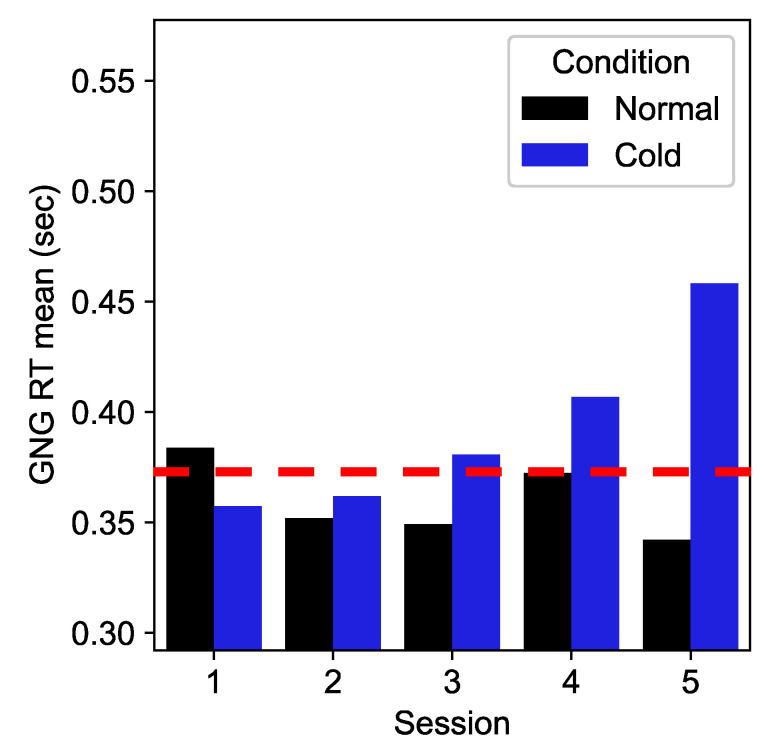
An example of a threshold for Subject 16’s GNG task. The red dashed line represents the threshold; the bars above this threshold line determine performance deteriorations. Sessions 3–5 are considered as “deteriorated in performance” since the blue bars for these sessions are above the threshold red line.

We obtained five segments from each subject, as each subject underwent five sessions during cold exposure. To account for the individual differences in cognitive task performance, individual thresholds were set to define performance deterioration per subject. Reaction time yielded in the cold condition that was slower than the 2nd slowest reaction time yielded in the normal condition (across all five sessions) was considered “deteriorated performance”, i.e., the threshold for deteriorated performance was equivalent to the 80th percentile of their baseline cognitive performance. Reaction times above the 80th percentile are considered reasonably slow, and this approach can remove outliers. Note that experimenters aimed to detect performance deterioration due to exposure only in the cold environment, as the normal environment condition was used to establish the threshold for performance deterioration. Figure 2 shows an example of finding a threshold to determine the number of deteriorated sessions in the GNG task for a given subject. In this example, cold sessions 3–5 were considered as “deteriorated in performance”, whereas sessions 1–2 were considered as “normal performance”.

### 2.5. Physiological Features

**Figure 3:**
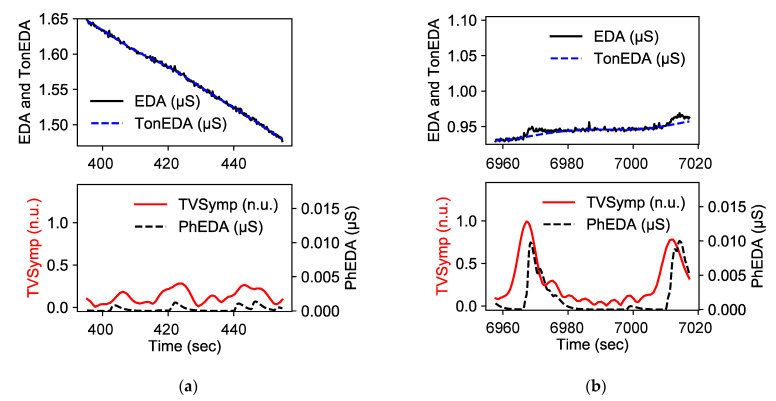
Example of EDA features: (**a**) normal session; (**b**) deteriorated session.

To detect performance deterioration in the cold condition, several features were derived from the EDA and ECG signals for each one-minute segment. Note that the one-minute EDA and ECG segments are aligned within the duration of each cognitive task (1.5 to 3 min). Although wearable devices were used to collect data, our visual inspection confirmed the absence of motion and noise artifacts, likely due to the well-controlled condition. The raw EDA signals were decomposed into phasic (PhEDA) and tonic (TonEDA) components using the cvxEDA technique [42]. Moreover, the time-varying index of sympathetic activity (TVSymp) was calculated. TVSymp computation is composed of two parts: (1) obtaining and reconstructing EDA signals in the frequency range between 0.08 and 0.24 Hz using variable frequency complex demodulation [43]; and (2) obtaining instantaneous amplitudes of the reconstructed signals using the Hilbert transform [44]. Note that the TVSymp computation process is fully described in a previous paper [32]. Figure 3 shows examples of EDA features.

For PhEDA, TonEDA, and TVSymp signals, we calculated the means and standard deviations from a one-minute segment centered in the middle of each task (0.5 min backward and forward from the midpoint). Furthermore, we calculated non-specific skin conductance responses (NSSCRs), which is the number of skin conductance responses in a period of time (i.e., one minute in our study) based on a threshold, normally set to be 0.05, from PhEDA [27]. Because cold acclimatization can decrease perspiration rates [45], we used two different thresholds (0.05 and 0.01 μS). EDA features are summarized in Table 3.

**Figure 4:**
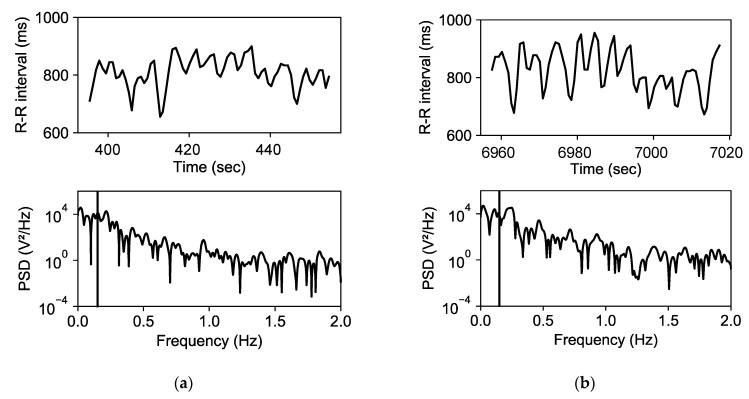
Example of HRV features: (**a**) normal session; (**b**) deteriorated session.

To calculate HRV features, we first detected peaks of QRS complexes using an approach based on the complete ensemble empirical mode decomposition [46,47]. Using R-peak intervals, we calculated high-frequency (HF) HRV, heart rate (HR), and the root mean square of successive differences (RMSSD) between normal heartbeats from each one-minute segment. We removed R-R intervals lower than 300 ms or greater than 1500 ms and ectopic beats using Malik’s rule [48]. The removed intervals were interpolated linearly. These features were calculated from the same one-minute segments as for EDA features. Figure 4 shows an example of R-R intervals and a power spectrum analysis. All HRV features are summarized in Table 3.

### 2.6. Statistics and Machine Learning

To statistically compare performance across sessions (i.e., exposure time) and conditions (normal and cold environments), we used a two-way (exposure time × condition) analysis of variance (ANOVA) on a linear mixed-effect model (R’s lmer function). For each cognitive performance metric, we investigated whether there was a significant effect of condition or any interactions between exposure time and the conditions [49,50]. Additionally, we used a *t*-test to compare physiological metrics between normal and deteriorated sessions. A *p*-value of < 0.05 was considered statistically significant.

We also examined machine learning (ML) algorithms to classify normal versus deteriorated sessions, using the extracted physiological features. The machine learning approaches consisted of support vector machines (SVMs) with a linear kernel, logistic regression (LR), random forest (RF), and multi-layer perceptron (MLP), using Python 3.9 with the Sckit-learn library 1.0.2. These algorithms have been widely used to automatically detect autonomic alteration [33,35,51].

We used leave-one-subject-out cross-validation to minimize the overfitting and procure subject independence of our results. For each fold, the following steps were conducted. First, SVM-Synthetic-Minority-Oversampling-Technique (SMOTE) was performed to make training datasets balanced [52]. Standardization was then conducted with a zero mean and unit variance, except for random forest. We tested machine learning models on EDA features, HRV features, and EDA + HRV features. In addition, we optimized the hyperparameters for each fold using only the training dataset. Hyperparameter selection is critical in determining a model’s ability to generalize, and incorrectly tuned hyperparameters may result in suboptimal model performance on unseen datasets [53]. In our study, hyperparameters of the machine learning algorithms were selected using the grid-search cross-validation with five-fold group cross-validation based on the geometric mean score, which is equivalent to the square root of multiplication of sensitivity and specificity:

(3) $$Geometric\;mean\;score=\sqrt{sensitivity\times specificity}$$

In each fold (i.e., one subject for testing and all the others for training), the training data are divided into five subject-based group folds. Then, all hyperparameter combinations are tested on the five subject-based group folds. Finally, a model with the hyperparameter combination that yields the highest geometric mean score is tested on the testing data. Thus, each fold used different hyperparameter combinations. The candidate hyperparameters are as follows. Parameter C for both SVM and LR was chosen from 0.01, 0.1, 1, 10, 100, and 1000. For RF, four different depths (3, 4, 5, and 6) were tested to calculate feature importance with the Gini criterion. Finally, MLP was tested with three different numbers of hidden layers (1, 2, and 3), with 100 hidden units, a rectifier linear unit activation function, the Adam optimizer, and a 0.001 learning rate. Note that training, validating, and testing data were completed separately. In addition to the geometric mean score, we calculated the area under the receiver operating characteristic curve (AUROC) to compare performance between models [54]. Additionally, we used the SHapley Additive exPlanations (SHAP) to evaluate the influence of each feature in terms of the degree of its contribution [55,56], which is a useful tool to evaluate feature importance of machine learning models using a game theory and related statistical methods.

## 3. Results

**Figure 5:**
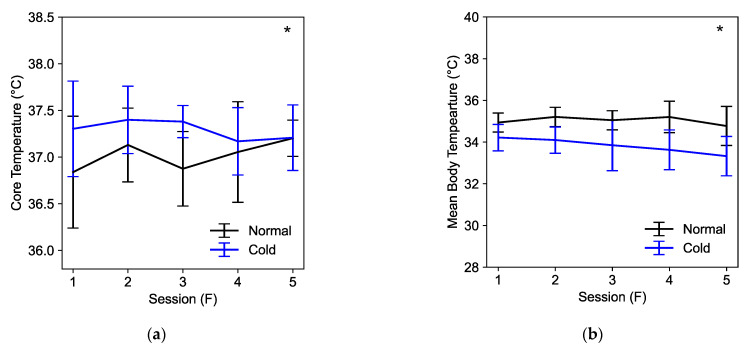
(**a**) Reaction time of the core temperature; (**b**) reaction time of the mean body temperature (mean ± SD). Asterisks represent significant differences in performance between normal and cold conditions.

Figure 5 shows a comparison of the core temperature and MBT between normal and cold conditions across five sessions. Our two-way ANOVA analysis detected marginally significant interactions in the core temperature between condition and exposure time (*F*(4,83) = 2.367, *p* = 0.059). MBT did not show significantly combined effect between condition and exposure time. A significant effect was observed between normal and cold sessions for both core temperature (*F*(1,91) = 11.679, *p* < 0.001) and MBT (*F*(1,89) = 72.162, *p* < 0.001).

**Figure 6:**
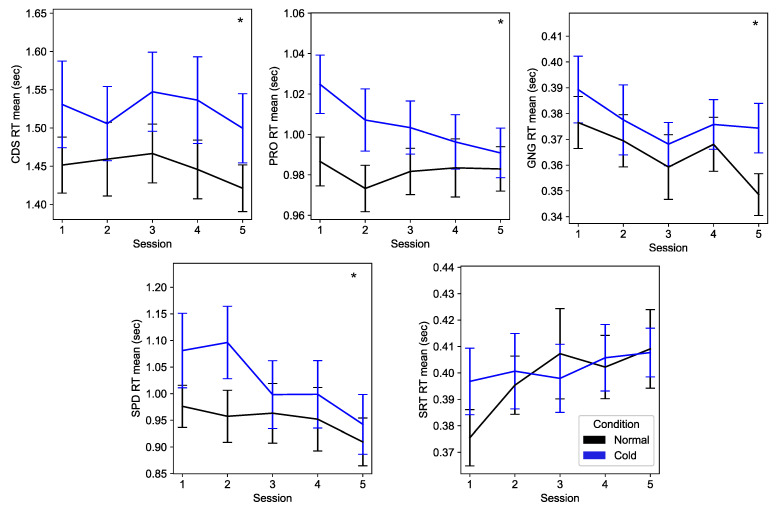
Reaction time of each cognitive task (mean ± SEM). Asterisks represent significant differences in performance between normal and cold conditions (*p* < 0.01).

Figure 6 shows a comparison of the reaction time of each cognitive task between normal and cold conditions across five sessions. Significant performance deterioration in the cold condition was observed during CDS (*F*(1,135) = 17.408, *p* < 0.001), PRO (*F*(1,162) = 27.847, *p* < 0.001), GNG (*F*(1,161) = 8.743, *p* = 0.004), and SPD (*F*(1,144) = 13.376, *p* < 0.001) compared to the normal condition. SRT did not show significant performance deterioration. Therefore, we excluded SRT in our analysis. Due to software errors during data collection, we excluded three and two subjects from CDS and SPD, respectively (Table 4). Our two-way ANOVA analysis did not detect significant interactions between condition and exposure time.

Table 5 shows the means and standard deviations (S.D.s) of EDA and HRV features between the normal and deteriorated sessions for each cognitive task. PhEDA’s mean and S.D. and TVSymp’s S.D. showed significant differences between normal and deteriorated performance in CDS and GNG (*p* < 0.05). TVSymp’s mean and NSSCR features showed significant differences in CDS only (*p* < 0.05). TonEDA features failed to detect any significant differences. Among HRV features, HR showed significant differences between normal and deteriorated performance in CDS, GNG, and SPD (*p* < 0.05), and RMSSD showed a significant difference in SPD (*p* < 0.05). HF HRV failed to detect any significant difference between normal and cold conditions. Notably, NSSCR 0.01 and HR in CDS, as well as HR in SPD, showed large effect sizes (Table 6).

We compared the geometric mean score (Table 7) and AUROC (Table 8) from four machine learning (ML) classifiers. For CDS, ML models trained with HRV features showed higher geometric mean scores than using EDA or EDA + HRV features. SVM showed the highest score, 0.824, with HRV features, while scores of 0.546 and 0.742 were achieved with EDA and EDA + HRV features, respectively. For CDS, AUROC values were also higher with HRV features (0.781–0.871), compared to EDA and EDA + HRV features. For PRO, the highest score was 0.568 with EDA + HRV features, obtained with RF. For GNG, the highest score of 0.696 was obtained using SVM with both EDA and HRV features. The highest AUROC value for GNG was also obtained using SVM with both EDA and HRV features. For SPD, the highest score was 0.620 with HRV features.

**Figure 7:**
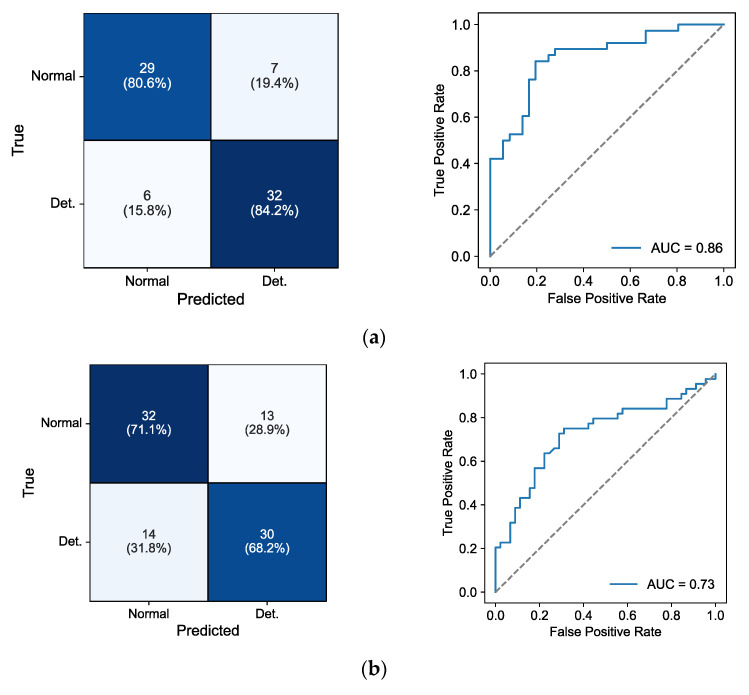
(**a**) Confusion matrices (left) and ROC curves (right) of SVM with HRV features for CDS; (**b**) confusion matrices (left) and ROC curves (right) of SVM with EDA + HRV features for GNG. Det—deteriorated session; AUC—area under the curve.

Figure 7 shows confusion matrices and the receiver operating characteristic (ROC) curves of CDS with HRV features and GNG with HRV + EDA features. CDS with HRV features showed an 82.4% accuracy with a sensitivity of 84.2% and a specificity of 80.6%. GNG with HRV + EDA features showed a 69.7% accuracy with a sensitivity of 68.2% and a specificity of 71.1%.

**Figure 8:**
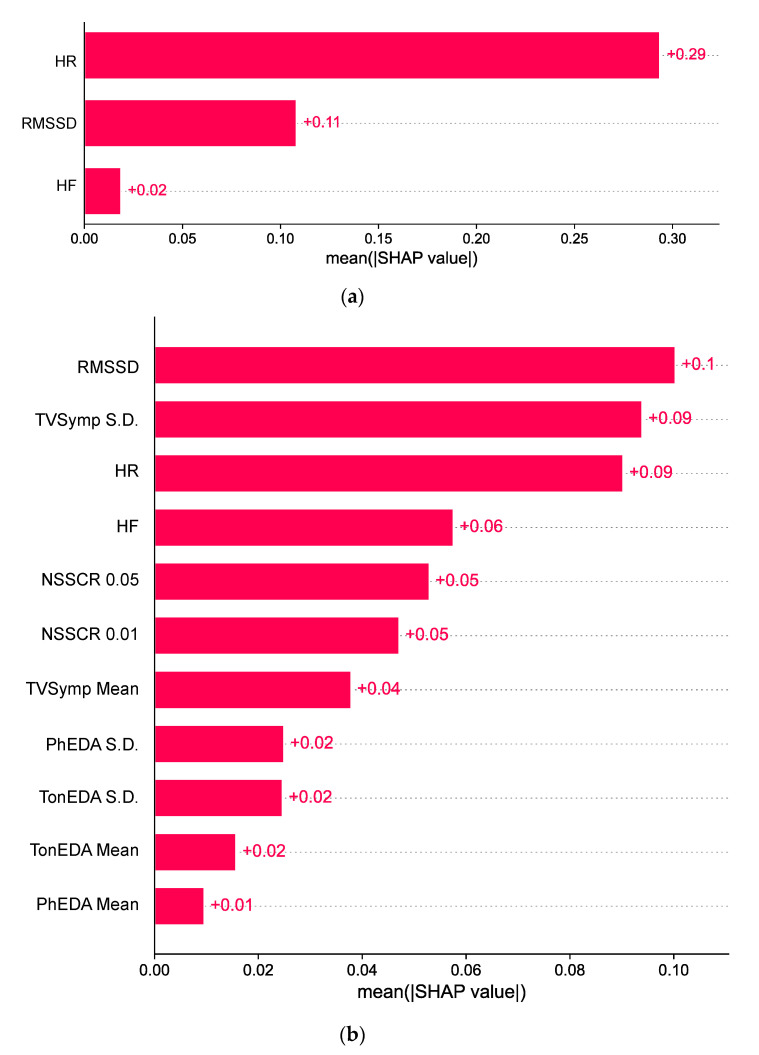
(**a**) SHAP analysis of SVM with HRV features for CDS; (**b**) SHAP analysis of SVM with EDA + HRV features for GNG.

Figure 8a,b show SHAP analyses on detecting performance deterioration in CDS using SVM with HRV features and in GNG using SVM with HRV + EDA features, respectively. Our SHAP analysis from the CDS model showed that HR was the most impactful feature, followed by RMSSD. HF was the least impactful feature for the classifier. For the analysis of GNG, we showed that HRV features were ranked at 1st, 2nd, and 4th in the most important features list. For EDA, TVSymp’s S.D. was the most important and ranked at the 3rd among all features, followed by NSSCR.

## 4. Discussion and Conclusions

We performed machine learning techniques on EDA- and HRV-derived features to detect cognitive performance deterioration due to cold exposure in female subjects. All cognitive tests showed impairment in reaction time, except for SRT. We tested machine learning classifiers on only reaction time because accuracies on these tasks did not show any deterioration during prolonged cold exposures. No impairment in accuracy but increased reaction time makes it unclear if the performance deterioration was elicited by cognitive impairment in the central nervous system, as evidenced by shivering response, inhibition of sweating, skin vasoconstriction, etc. [3], or by generally slower bodily movement caused by reduced blood flow due to the prolonged exposure to the cold environment. Studies showed that females generally experience lower blood flow in their extremities when exposed to cold, due to increased vascular reactivity [57]. Our presumption is that the combination of those is responsible for the slower reaction time in the cognitive tests, because the simple reaction time (SRT) task did not differ between normal and cold environments.

Our results showed up to 82.4% of the geometric mean score for code substitution (CDS), which is associated with short-term memory. The other tasks showed lower scores: 69.6% for Go/No-go (GNG), 62.0% for spatial discrimination (SPD), and 56.8% for procedural reaction time (PRO). For CDS, machine learning models with HRV showed higher performance than models with EDA or EDA + HRV features. In addition, HRV showed higher SHAP values than EDA features, indicating the former’s greater impact in the machine learning models. This is likely due to the complexity of sweat gland responses to cold exposure. EDA is primarily mediated by acetylcholine [27] to which muscarinic receptors respond. Muscarinic receptors are primarily associated with the parasympathetic nervous system, except where they stimulate the sweat glands under the control of the SNS [58]. However, increased skin sympathetic nerve activity during cold exposure is thought to be primarily noradrenergic [59], which is also shown in a rat study [60]. Thus, EDA signals may be attenuated during cold exposures compared to those recorded at normal temperature, as shown in our previous study where decreased EDA amplitudes were observed [7]. Additionally, several studies have demonstrated changes in HRV indices during cold exposure, such as increased LF and vLF [17,18,20]. Nevertheless, TVSymp was the 2nd most influential feature based on SHAP analysis for GNG. TVSymp also showed high sensitivity in detecting thermal stimulus, electric shock stimulus, and dental pain [33,34,61]. Moreover, the mean and S.D. of phasic and tonic components of EDA were found to be the least influential features in the analysis.

There have been several previous studies to detect cognitive performance deterioration using HRV and/or EDA [24,25,26,36,37]. For instance, a study showed the feasibility of detecting performance deterioration via the measure of reaction time due to prolonged wakefulness [36]. In that study [36], EDA showed higher classification power than HRV, while in this study HRV features demonstrated increased classification accuracy than EDA features. We hypothesize that this is because sleep deprivation is more associated with increased SNS activity [62], while cold environment is associated with increased PNS activity [17]. Additionally, other physiological measurements have been utilized to detect performance deterioration. For example, studies have demonstrated the feasibility of detecting performance deterioration during prolonged wakefulness via reaction time using a webcam and/or eye-tracking glasses [63,64]. However, a webcam can only be used in specific environments where subjects remain stationary, and eye-tracking glasses can be intrusive due to head movements and other visual distractions, making it more difficult to adopt these into real-world scenarios. Moreover, a webcam requires that neither clothing nor extensive equipment be on the face, which is difficult for cold operational environments in which individuals must adorn extensive gear. Therefore, noninvasive EDA and HRV that work within the limitations and requirements of real-world cold environments are promising approaches to detecting performance deterioration due to prolonged cold exposure.

Overall, our result suggests that cognitive performance deterioration due to cold exposure, especially in a task utilizing short-term memory, can be effectively detected using commonly available wearable devices such as those that collect ECG and EDA. Our models can be used for application to female military personnel deployed in cold environments, enabling preemptive action to reduce risk during operations and facilitating the development of individualized, situation-dependent scheduling and shift rotation strategies.

### Limitations and Future Directions

Despite our promising results, the current study only assessed aspects of cognitive performance conducted during limited environmental exposure durations (140 min), with one of the environmental conditions being a 10 °C cold environment. In future studies, longer exposure times and even lower temperatures should be considered. Sex-based response to immersion in cold water should also be explored for scientific and operational reasons. In addition, females with different body mass index (BMI) and body fat groups may have varying autonomic nervous system (ANS) responses in a cold environment, which could result in different outcomes with our machine learning models [20].

Moreover, motion artifact noise is another issue when using wearable devices. The presence of motion artifact noise in EDA and ECG signals, which can occur due to unstable skin−electrode contact, muscular activity, movement, and other factors, can increase the likelihood of false decisions, leading to inaccurate classification results [65,66]. In our study, we did not observe noticeable motion artifact noise, as the data were collected in a well-controlled environment. When our models are implemented in the wearable systems, motion artifact detection and removal algorithms for ECG and EDA signals must be implemented to minimize potential false positives and negatives [67,68,69,70,71,72].

Another limitation is the potential impact of caffein withdrawal, which could affect reaction times in our cognitive battery tasks [73]. In our study, we asked participants to refrain from caffeine prior to each experiment, because caffeine consumption can affect both reaction time [74] and the autonomic nervous system [75]. Future studies should consider broader confounders, including habitual stimulant use and varying levels of stress.

Regarding machine learning, the models were trained using only a small sample size with a non-diverse population (female undergraduate and graduate students). Therefore, our models may not perform well when tested on other groups, such as military personnel. Future studies should train and test machine learning models using more diverse data to improve the generalizability of the models, including mixed gender, diverse age, and diverse body composition (e.g., BMI). In addition, future studies should investigate diverse environmental stressors such as colder temperatures, longer exposure to cold, heat stress, and hypoxia. In addition, multimodal data fusion should be tested by incorporating additional physiological sensors, such as temperature and electromyography. Furthermore, future studies, especially those using a new dataset with diverse population and multimodal sensors, should explore feature selection and dimensionality reduction schemes, such as principal component analysis [76]. In our study, we did not apply any feature selection or dimensionality reduction scheme due to the small number of data and features.

Finally, in real-world scenarios, wearable EDA and ECG devices face challenges related to usability and comfortableness. To implement our models effectively, EDA and ECG sensors need to be well designed for long-term monitoring, ensuring usability and comfortableness without disrupting physical activity. These factors are critical as they can impact the models’ performance. In addition, it is noteworthy that the time complexity of our SVM models, which achieved the highest geometric mean scores, is *O(d)* where *d* denotes the number of features. Therefore, both computation time and memory requirements are relatively efficient. However, the development and implementation of memory- and computation-efficient techniques for ECG motion artifact detection and removal, ECG peak detections, and HRV feature calculation present significant challenges. This should be investigated in future studies.

## Figures and Tables

**Table 1:** Summary of study procedures.

	Day 1	Day 2	Cumulative exposure time
Demographic questionnaire	O		
Two practice sets of the cognitive task battery	O		
Baseline	5 min	5 min	5 min
Session1	15 min	15 min	20 min
Break	15 min	15 min	35 min
Session 2	15 min	15 min	50 min
Break	15 min	15 min	65 min
Session 3	15 min	15 min	80 min
Break	15 min	15 min	95 min
Session 4	15 min	15 min	110 min
Break	15 min	15 min	125 min
Session 5	15 min	15 min	140 min

Each subject was exposed to either a normal or cold environment on different days. The orders of conditions and tasks were randomized.

**Table 2:** Cognitive performance.

Task	Duration (min)	Evaluation
Code substitution (CDS)	2	Short-term memory and attention
Procedural reaction time (PRO)	1.5	Ability to accurately respond while inhibiting premature responses
Go/No-go (GNG)	2.5	Omission and commission
Spatial discrimination (SPD)	3	Spatial manipulation
Simple reaction time (SRT)	1.5	Attention

**Table 3:** Summary of physiological features.

Features	Description	Remarks
	EDA	
PhEDA	Phasic component of EDA obtained using the cvxEDA decomposition method	Mean, S.D.
TonEDA	Tonic component of EDA obtained using the cvxEDA decomposition method	Mean, S.D.
TVSymp	Reconstructed EDA signals in the frequency range between 0.08 and 0.24 Hz	Mean, S.D.
NSSCR 0.05	The number of skin conductance response greater than 0.05 µS	
NSSCR 0.01	The number of skin conductance response greater than 0.01 µS	
	HRV	
HF	Absolute power of the high-frequency band (0.15–0.4 Hz)	
HR	The number of heart beats per minute	
RMSSD	The root mean square of successive differences between normal heartbeats	

**Table 4:** Number of subjects and segments available for both HRV and EDA features per each task.

Task	Subjects	Normal	Deteriorated
CDS	16	36	38
PRO	19	33	54
GNG	19	45	44
SPD	17	37	42

**Table 5:** EDA and HRV features between normal performance and deteriorated performance sessions (mean ± S.D. with 95% confidence intervals).

	CDS		PRO		GNG		SPD	
	Normal	Det.	Normal	Det.	Normal	Det.	Normal	Det.
EDA								
PhEDA Mean	0.01 ± 0.02 (0.00–0.01)	0.06 ± 0.10 * (0.03–0.10)	0.04 ± 0.12 (−0.00–0.08)	0.03 ± 0.06 (0.02–0.05)	0.01 ± 0.02 (0.00–0.02)	0.03 ± 0.08 * (0.01–0.06)	0.06 ± 0.20 (−0.01–0.12)	0.05 ± 0.13 (0.01–0.09)
PhEDA S.D.	0.01 ± 0.03 (0.00–0.02)	0.06 ± 0.09 * (0.03–0.09)	0.03 ± 0.06 (0.01–0.05)	0.03 ± 0.06 (0.02–0.05)	0.01 ± 0.03 (0.01–0.02)	0.04 ± 0.08 * (0.02–0.06)	0.06 ± 0.21 (−0.01–0.13)	0.05 ± 0.10 (0.02–0.09)
TonEDA Mean	6.23 ± 4.62 (4.67–7.79)	8.39 ± 5.19 (6.69–10.10)	8.01 ± 5.34 (6.12–9.90)	7.37 ± 5.45 (5.89–8.86)	6.97 ± 4.97 (5.48–8.47)	8.15 ± 5.45 (6.49–9.80)	8.77 ± 5.07 (7.08–10.46)	7.16 ± 5.01 (5.60–8.72)
TonEDA S.D.	0.09 ± 0.10 (0.06–0.13)	0.14 ± 0.13 (0.10–0.18)	0.13 ± 0.13 (0.08–0.18)	0.13 ± 0.15 (0.09–0.17)	0.14 ± 0.14 (0.10–0.18)	0.18 ± 0.18 (0.13–0.24)	0.22 ± 0.48 (0.06–0.38)	0.13 ± 0.12 (0.09–0.17)
TVSymp Mean	0.10 ± 0.12 (0.06–0.14)	0.27 ± 0.41 * (0.13–0.40)	0.22 ± 0.31 (0.11–0.33)	0.30 ± 0.57 (0.15–0.46)	0.11 ± 0.12 (0.08–0.15)	0.19 ± 0.27 (0.11–0.28)	0.25 ± 0.54 (0.07–0.43)	0.31 ± 0.52 (0.14–0.47)
TVSymp S.D.	0.08 ± 0.14 (0.03–0.12)	0.19 ± 0.27 * (0.10–0.28)	0.17 ± 0.23 (0.09–0.25)	0.22 ± 0.39 (0.11–0.32)	0.08 ± 0.10 (0.05–0.11)	0.18 ± 0.27 * (0.09–0.26)	0.23 ± 0.44 (0.08–0.38)	0.28 ± 0.46 (0.14–0.42)
NSSCR 0.05	0.33 ± 1.20 (−0.07–0.74)	2.61 ± 3.97 * (1.30–3.91)	2.24 ± 4.06 (0.81–3.68)	1.76 ± 2.97 (0.95–2.57)	1.00 ± 2.32 (0.30–1.70)	1.96 ± 3.20 (0.98–2.93)	1.81 ± 3.37 (0.69–2.93)	1.83 ± 3.08 (0.87–2.79)
NSSCR 0.01	1.72 ± 2.91 (0.74–2.71)	4.95 ± 4.05 * (3.62–6.28)	4.36 ± 4.54 (2.75–5.97)	3.24 ± 3.41 (2.31–4.17)	2.53 ± 3.71 (1.42–3.65)	3.84 ± 4.15 (2.58–5.10)	4.57 ± 4.22 (3.16–5.98)	3.67 ± 3.85 (2.47–4.87)
HRV								
HR	70.4 ± 6.5 (68.2–72.6)	82.3 ± 10.9 * (78.8–85.9)	75.2 ± 10.4 (71.5–78.9)	77.4 ± 8.4 (75.1–79.6)	71.7 ± 8.4 (69.2–74.2)	77.9 ± 9.4 * (75.1–80.8)	71.4 ± 7.9 (68.8–74.1)	79.6 ± 10.0 * (76.5–82.7)
RMSSD	47.1 ± 12.1 (43.0–51.2)	43.7 ± 20.3 (37.0–50.4)	45.3 ± 16.2 (39.6–51.1)	42.3 ± 17.8 (37.5–47.2)	49.2 ± 18.1 (43.7–54.7)	49.5 ± 16.8 (44.4–54.7)	51.5 ± 14.5 (46.7–56.4)	44.2 ± 15.2 * (39.4–48.9)
HF	648 ± 469 (490–807)	894 ± 1029 (556–1232)	702 ± 656 (469–934)	595 ± 560 (442–747)	867 ± 749 (642–1092)	832 ± 602 (649–1014)	874 ± 612 (670–1079)	797 ± 702 (578–1015)

Asterisks indicate significant differences between normal and deteriorated sessions (at *p* < 0.05; *t*-test).

**Table 6:** EDA and HRV features between normal performance and deteriorated performance sessions (*p*-value and Cohen’s *d*).

	CDS		PRO		GNG		SPD	
	*p*-Value	Cohen’s *d* (95%CI)	*p*-Value	Cohen’s *d* (95%CI)	*p*-Value	Cohen’s *d* (95%CI)	*p*-Value	Cohen’s *d* (95%CI)
PhEDA Mean	0.002 *	0.74 (0.27–1.21)	0.685	−0.09 (−0.52–0.34)	0.047 *	0.43 (0.01–0.85)	0.911	−0.03 (−0.47–0.42)
PhEDA S.D.	0.004 *	0.70 (0.23–1.17)	0.976	0.01 (−0.43–0.44)	0.028 *	0.47 (0.05–0.89)	0.826	−0.05 (−0.49–0.39)
TonEDA Mean	0.063	0.44 (−0.02–0.90)	0.594	−0.12 (−0.55–0.32)	0.292	0.23 (−0.19–0.64)	0.161	−0.32 (−0.76–0.13)
TonEDA S.D.	0.07	0.43 (−0.03–0.89)	0.938	0.02 (−0.42–0.45)	0.254	0.24 (−0.17–0.66)	0.226	−0.28 (−0.72–0.17)
TVSymp Mean	0.024 *	0.54 (0.07–1.00)	0.419	0.18 (−0.26–0.61)	0.075	0.38 (−0.04–0.80)	0.657	0.1 (−0.34–0.54)
TVSymp S.D.	0.025 *	0.53 (0.07–1)	0.49	0.15 (−0.28–0.59)	0.022 *	0.49 (0.07–0.92)	0.621	0.11 (−0.33–0.55)
NSSCR 0.05	0.002 *	0.77 (0.29–1.24)	0.524	−0.14 (−0.58–0.29)	0.11	0.34 (−0.08–0.76)	0.975	0.01 (−0.44–0.45)
NSSCR 0.01	<0.001	**0.91** (0.43–1.39)	0.193	−0.29 (−0.73–0.15)	0.12	0.33 (−0.09–0.75)	0.324	−0.22 (−0.67–0.22)
HR	<0.001	**1.328** (0.83–1.83)	0.292	0.23 (−0.20–0.67)	0.001	0.70 (0.27–1.13)	<0.001 *	**0.9** (0.44–1.36)
RMSSD	0.383	−0.20 (−0.66–0.25)	0.435	−0.17 (−0.61–0.26)	0.927	0.02 (−0.40–0.44)	0.031 *	−0.50 (−0.94–−0.05)
HF	0.195	0.30 (−0.15–0.76)	0.421	−0.18 (−0.61–0.26)	0.806	−0.05 (−0.47–0.36)	0.603	−0.12 (−0.56–0.32)

Bold fonts indicate the Cohen’s d values greater than 0.80 (i.e., large effect sizes). Asterisks indicate significant differences between normal and deteriorated sessions (at *p* < 0.05; *t*-test).

**Table 7:** Geometric mean score of classifiers (95% confidence intervals).

	Features	LR	SVM	RF	MLP
CDS	EDA	0.579 (0.480–0.790)	0.546 (0.385–705)	0.621 (0.465–778)	0.635 (0.480–790)
	HRV	0.784 (0.619–0.895)	**0.824** (0.701–946)	0.743 (0.603–884)	0.757 (0.619–895)
	Both	0.729 (0.540–0.838)	0.742 (0.601–883)	0.647 (0.492–801)	0.689 (0.540–838)
PRO	EDA	0.402 (0.231–0.548)	0.424 (0.260–577)	0.52 (0.357–674)	0.396 (0.231–548)
	HRV	0.318 (0.136–0.446)	0.379 (0.211–530)	0.437 (0.276–591)	0.302 (0.136–446)
	Both	0.355 (0.246–0.568)	0.452 (0.296–605)	**0.568** (0.406–720)	0.414 (0.246–568)
GNG	EDA	0.516 (0.475–0.761)	0.525 (0.378–672)	0.558 (0.411–703)	0.618 (0.475–761)
	HRV	0.685 (0.533–0.808)	0.662 (0.523–801)	0.58 (0.435–725)	0.671 (0.533–808)
	Both	0.651 (0.485–0.770)	**0.696** (0.561–831)	0.584 (0.439–729)	0.628 (0.485–770)
SPD	EDA	0.45 (0.388–0.700)	0.481 (0.323–638)	0.414 (0.259–568)	0.544 (0.388–700)
	HRV	**0.62** (0.415–0.724)	0.583 (0.429–737)	0.479 (0.323–635)	0.569 (0.415–724)
	Both	0.559 (0.342–0.657)	0.553 (0.397–708)	0.532 (0.376–688)	0.501 (0.342–657)

Bold fonts indicate the highest geometric mean scores for each cognitive task.

**Table 8:** AUROCs of classifiers (95% confidence intervals).

	Features	LR	SVM	RF	MLP
CDS	EDA	0.6 (0.469–0.730)	0.673 (0.551–0.796)	0.645 (0.517–0.760)	0.677 (0.548–0.793)
	HRV	**0.871** (0.781–0.941)	0.857 (0.767–0.934)	0.781 (0.673–0.877)	0.848 (0.754–0.928)
	Both	0.789 (0.676–0.886)	0.779 (0.663–0.875)	0.744 (0.623–0.848)	0.753 (0.623–0.857)
PRO	EDA	0.424 (0.303–0.549)	0.443 (0.321–0.572)	0.513 (0.382–0.646)	0.338 (0.224–0.457)
	HRV	0.246 (0.139–0.362)	0.311 (0.178–0.446)	0.422 (0.304–0.545)	0.253 (0.157–0.365)
	Both	0.375 (0.260–0.500)	0.275 (0.154–0.393)	**0.546** (0.412–0.677)	0.395 (0.275–0.530)
GNG	EDA	0.589 (0.469–0.706)	0.517 (0.387–0.635)	0.567 (0.446–0.678)	0.609 (0.487–0.725)
	HRV	0.715 (0.602–0.826)	0.7 (0.586–0.807)	0.587 (0.466–0.699)	0.724 (0.617–0.825)
	Both	0.663 (0.542–0.777)	**0.727** (0.612–0.827)	0.542 (0.417–0.656)	0.652 (0.526–0.764)
SPD	EDA	0.335 (0.208–0.460)	0.406 (0.268–0.526)	0.426 (0.304–0.560)	0.620 (0.489–0.746)
	HRV	**0.633** (0.501–0.757)	0.573 (0.443–0.708)	0.551 (0.418–0.682)	0.616 (0.487–0.744)
	Both	0.606 (0.485–0.736)	0.495 (0.366–0.627)	0.546 (0.422–0.671)	0.532 (0.409–0.658)

Bold fonts indicate the highest geometric mean scores for each cognitive task.

## Data Availability

The raw data supporting the conclusions of this article will be made available by the authors on request.

## Appendix A

**Table A1:** Weight adjustment for ISO 9886:2004.

Sites	8-Point Weights (ISO 9886:2004)		3-Point Weights (Our Study)
Forehead	0.07	×3.125	0.21875
Right scapula	0.175		
Left upper chest	0.175		
Right arm in an upper location	0.07		
Left arm in a lower location	0.07		
Left hand	0.05	×3.125	0.15625
Right anterior thigh	0.19		
Left calf	0.2	×3.125	0.625
Summation	1.00		1.00
